# TRP Channels Involved in Spontaneous l-Glutamate Release Enhancement in the Adult Rat Spinal Substantia Gelatinosa

**DOI:** 10.3390/cells3020331

**Published:** 2014-04-29

**Authors:** Eiichi Kumamoto, Tsugumi Fujita, Chang-Yu Jiang

**Affiliations:** Department of Physiology, Saga Medical School, 5-1-1 Nabeshima, Saga 849-8501, Japan; E-Mails: fujitat@cc.saga-u.ac.jp (T.F.); jiang-changyu@hotmail.com (C.-Y.J.)

**Keywords:** TRPV1 channel, TRPA1 channel, excitatory transmission, inhibitory transmission, spinal dorsal horn, pain

## Abstract

The spinal substantia gelatinosa (SG) plays a pivotal role in modulating nociceptive transmission through dorsal root ganglion (DRG) neurons from the periphery. TRP channels such as TRPV1 and TRPA1 channels expressed in the SG are involved in the regulation of the nociceptive transmission. On the other hand, the TRP channels located in the peripheral terminals of the DRG neurons are activated by nociceptive stimuli given to the periphery and also by plant-derived chemicals, which generates a membrane depolarization. The chemicals also activate the TRP channels in the SG. In this review, we introduce how synaptic transmissions in the SG neurons are affected by various plant-derived chemicals and suggest that the peripheral and central TRP channels may differ in property from each other.

## 1. Introduction

Nociceptive stimuli given to the periphery generate a membrane depolarization in the peripheral terminals of primary-afferent, particularly fine myelinated Aδ and unmyelinated C, fibers, resulting in the production of action potentials (APs). The APs conduct through the primary-afferent fibers to the central terminals located in the superficial laminae of the dorsal horn, especially the substantia gelatinosa (SG; lamina II of Rexed; [1,2]). As a result, excitatory neurotransmitter l-glutamate is mono- or polysynaptically released onto the SG neurons from the central terminals [3]. Such nociceptive information is modulated by a neuronal circuitry composed of excitatory and inhibitory interneurons in the SG ([3,4]; see [5] for a role of inhibitory interneurons in nociceptive transmission) and by the descending antinociceptive pathway. Information as a result of the modulation flows to the thalamus through a connection with projection neurons in lamina I and deeper laminae of the spinal dorsal horn, and then to the primary sensory area of the cerebral cortex, eliciting nociceptive sensation (for review see [6,7]).

Since Melzack and Wall [8] proposed the gate control theory of pain, a great deal of evidence has been demonstrated to support a role of the SG in the modulation of nociceptive transmission. For instance, endogenous analgesics such as opioids, nociceptin, serotonin, norepinephrine, adenosine, and galanin reduce the release of l-glutamate from the central terminals of primary-afferent fibers onto the SG neurons through the activation of their receptors, resulting in diminishing the excitability of the neurons (for example see [9,10,11,12,13,14]; for review see [15]). Moreover, activation of receptors for serotonin, norepinephrine and also another analgesic acetylcholine enhances the release of inhibitory neurotransmitters, GABA and/or glycine, onto the SG neurons, leading to an inhibition of the excitability of the neurons ([16,17,18,19]; also see [20]).

**Figure 1:**
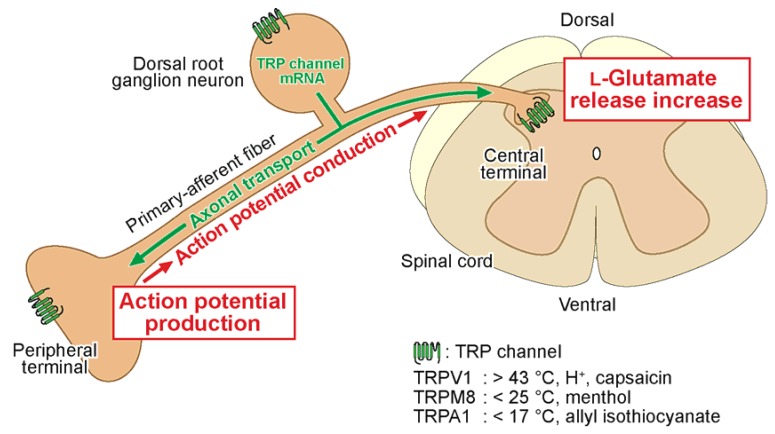
Schematic diagram illustrating what roles transient receptor potential (TRP) channels in the peripheral and central terminals of dorsal root ganglion (DRG; primary-afferent) neuron play in transmitting sensory information. The TRP channels are synthesized in the cell body of the DRG neuron and transferred to the terminals by axonal transport. Stimuli (such as temperature and chemicals) given to the periphery activate the peripheral TRP channel, resulting in membrane depolarization, which in turn generates action potential that transfers the stimulus information to the central terminal of the DRG neuron. On the other hand, central TRP activation enhances the spontaneous release of l-glutamate onto spinal substantia gelatinosa (SG) neurons, which play a pivotal role in modulating nociceptive transmission and is thus involved in this modulation.

There are not only neurotransmitter receptors but also transient receptor potential (TRP) channels (which are permeable to cations) among proteins involved in the modulation of nociceptive transmission (for a review, see [21]). TRP channels, which are synthesized in the cell body of dorsal root ganglion (DRG) neuron, are transferred to the peripheral and central terminals of the neuron by axonal transport (Figure 1). There are TRP vanilloid-1 (TRPV1), TRP ankyrin-1 (TRPA1) and TRP melastatin-8 (TRPM8) channels among TRP channels involved in nociception in DRG neurons [22]; they respond to chemical substances and temperature (for review see [23]). In the peripheral terminal of the DRG neuron, the TRPV1 channel responds to capsaicin (a natural pungent ingredient in red peppers), protons and noxious heat (>43 °C; [24]; for a review, see [25]); the TRPA1 channel to pungent compounds in mustard oil, cinnamon oil, ginger and garlic, and to noxious cold temperature (<17 °C; [26,27,28,29]); and the TRPM8 channel to menthol (2-isopropyl-5-methylcyclohexanol; a secondary alcohol which is contained in peppermint or other mint oils) and mild temperature (<25 °C; [30,31]). With respect to the TRPA1 channel, its cold sensitivity remains controversial and its involvement in cold hypersensitivity, but not nociception, has been demonstrated by del Camino *et al.* [32]. Many of the properties of the TRP channels have been examined in the cell body of DRG neuron and in heterologous cells expressing the TRP channels. It is possible that there is a difference in property between TRP channels in the peripheral and central terminals of DRG neuron, considering their distinct roles in the terminals.

This review article will introduce our data about the actions of plant-derived chemicals having an ability to activate TRP channels on synaptic transmissions in the SG neurons of adult rat spinal cord slices.

## 2. Spinal Cord Slice and Electrophysiology

The methods used for obtaining transverse slice preparations of the adult rat spinal cord have been mentioned previously in detail [33]. Briefly, male Sprague-Dawley rats (6–8 week-old) were anesthetized with urethane, and then a lumbosacral segment (L1–S3) of the spinal cord was removed and placed in oxygenated and cold Krebs solution. After cutting all ventral and dorsal roots, the pia-arachnoid membrane was removed. The spinal cord was placed in a shallow groove formed in an agar block, and this was mounted on a stage in a microslicer, which was filled with preoxygenated cold Krebs solution; then about 650 µm-thick transverse slice was cut. Adult rat spinal cord slice that retained an attached dorsal root was also used. The slice was placed on a nylon mesh in the recording chamber [34], and was then completely submerged and superfused at a rate of 10–15 mL/min with heated and oxygenated Krebs solution. The composition of Krebs solution used was (in mM): NaCl, 117; KCl, 3.6; CaCl_2_, 2.5; MgCl_2_, 1.2; NaH_2_PO_4_, 1.2; NaHCO_3_, 25; and glucose, 11.

SG neurons were identified by their location under a binocular microscope with light transmitted from below. Blind whole-cell voltage-clamp recordings were made from the SG neurons, as mentioned previously [35]. The patch-pipette solution used contained (in mM): K-gluconate, 135; KCl, 5; CaCl_2_, 0.5; MgCl_2_, 2; EGTA, 5; HEPES, 5; and Mg-ATP, 5; or Cs_2_SO_4_, 110; CaCl_2_, 0.5; MgCl_2_, 2; EGTA, 5; HEPES, 5; Mg-ATP, 5; tetraethylammonium (TEA)-Cl, 5. The former and latter solutions were used to record excitatory and inhibitory postsynaptic currents (EPSCs and IPSCs, respectively), respectively. A liquid junction potential between the gluconate (or Cs^+^)-containing patch-pipette and Krebs solutions was 12.4 mV (or 10.7 mV). EPSCs were recorded at a holding potential (V_H_) of −70 mV, where no IPSCs were observed, since the reversal potential for IPSCs was near −70 mV. On the other hand, IPSCs were observed at a V_H_ of 0 mV, where EPSCs were invisible owing to the reversal potential for EPSCs to be close to 0 mV. Cs^+^ and TEA were added to inhibit K^+^ channels located in the recorded SG neurons and thus to easily shift V_H_ to 0 mV from resting membrane potentials. Aδ-fiber and C-fiber evoked EPSCs were elicited by stimulating the dorsal root, as mentioned previously [34]. Their evoked EPSCs were distinguished from each other, based on a minimal stimulus strength enough to elicit the EPSCs and a latency of the EPSCs. Aδ-fiber EPSCs were judged to be monosynaptic when the latency remained constant and there was no failure during stimulation at 20 Hz for 1 s, while C-fiber ones were so when failures did not occur during repetitive stimulation at 1 Hz for 20 s [34,36]. Signals were acquired using a patch-clamp amplifier. Drugs were applied by perfusing a solution containing drugs of a known concentration without an alteration in the perfusion rate and temperature. The solution in the recording chamber having a volume of 0.5 mL was completely replaced within 15 s.

## 3. Actions of Plant-Derived TRP Agonists on Synaptic Transmissions in Substantia Gelatinosa Neurons

### 3.1. Action of Capsaicin

**Figure 2:**
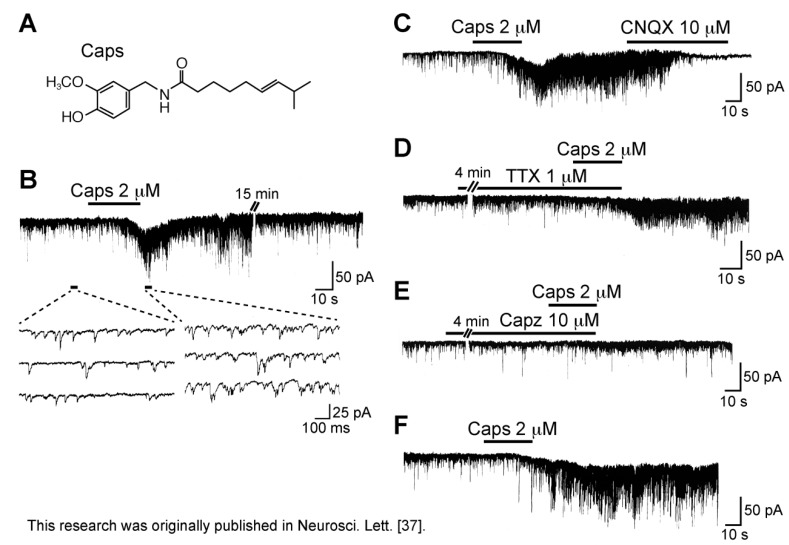
Capsaicin (Caps; 2 µM) increases the frequency of spontaneous excitatory postsynaptic current (EPSC) and produces an inward current in rat substantia gelatinosa (SG) neurons by activating TRPV1 channels. (**A**) The chemical structure of Caps; (**B**) Recordings of spontaneous EPSCs (sEPSCs) before and under the action of Caps. In this and subsequent figures, the horizontal bar above the chart recording indicates the period of time during which the drug was applied, and 3–4 consecutive traces of sEPSCs for a period indicated by a short bar below the recording are shown in an expanded time scale; (**C**) Action of a non-NMDA receptor antagonist CNQX (10 µM) on a barrage of sEPSCs under the action of Caps; (**D**) The sEPSC frequency increase produced by Caps is unaffected by a voltage-gated Na^+^-channel blocker tetrodotoxin (TTX; 1 µM); (**E**) Action of Caps in the presence of a TRPV1 antagonist capsazepine (Capz; 10 µM); Note that the sEPSC frequency increase as well as inward current were markedly attenuated by Capz; (**F**) Caps action at 15 min after washout of Capz, where sEPSC frequency was increased and an inward current lasting for 3 min (shown partly) was induced; this was obtained from the same neuron as that in (**E**). Holding potential (V_H_) was −70 mV. Modified from [37] with permission of Elsevier Science.

Superfusing capsaicin (8-methyl-*N*-vanillyl-6-nonenamide; Figure 2A) at 2 µM for 0.5 min enhanced the frequency of spontaneous EPSC (sEPSC) in SG neurons, as seen in Figure 2B. This increase in sEPSC frequency was about 234%; this action was almost irreversible such that this was not observed even 2 h after the capsaicin treatment. A non-*N*-methyl-d-aspartate (non-NMDA) receptor antagonist 6-cyano-7-nitroquinoxaline-2,3-dione (CNQX) at 10 µM blocked sEPSCs under the action of capsaicin (see Figure 2C), indicating that these sEPSCs were glutamatergic [37]. Since a voltage-gated Na^+^-channel blocker tetrodotoxin (TTX; 1 µM) did not affect the capsaicin activity, this was due to a direct action of capsaicin (Figure 2D). As seen in Figure 2E, in the presence of a TRPV1 antagonist capsazepine (10 µM; [38]), capsaicin (2 µM) had no effect on sEPSC frequency, indicating an involvement of TRPV1 channel [37,39,40]. This result is consistent with the existence of TRPV1 channels in the central terminals of primary-afferent neurons [41,42,43]. Ueda *et al.* [44] have demonstrated a TTX-insensitive and capsazepine-sensitive increase of l-glutamate release evoked by capsaicin by using a fluorometric monitoring method in slices prepared from the dorsal horn of the rat spinal cord. A similar action of capsaicin has been reported in the superficial medullary dorsal horn [45]. The sEPSC frequency increase would be due to the facts that capsaicin opens TRPV1 channels having a higher Ca^2+^ permeability and that the resulting depolarization activates voltage-gated Ca^2+^ channels [24,46]. Medvedeva *et al.* [47] have demonstrated that TRPV1 activation by capsaicin at synapses in DRG/spinal cord co-cultures prolongs the elevation of intraterminal Ca^2+^ levels and increases l-glutamate release.

On the other hand, Baccei *et al.* [39] have reported that capsaicin-induced sEPSC frequency increase persists in a nominally Ca^2+^-free solution in neonate rat superficial dorsal horn neurons, indicating that this increase does not require Ca^2+^ entry from extracellular solution. Although this issue needs further study, TRPV1 channels may alter in property with development.

Unlike glutamatergic transmission, spontaneous GABAergic and glycinergic transmissions, which were examined in the presence of a glycine-receptor antagonist strychnine (1 µM) and a GABA_A_-receptor antagonist bicuculline (10 µM), respectively, were not affected by capsaicin (2 µM; [37]). As a result, TRPV1 channels were suggested to be not located in the central terminals of primary-afferent fibers innervating onto spinal inhibitory interneurons, which make synapses with SG neurons.

**Figure 3:**
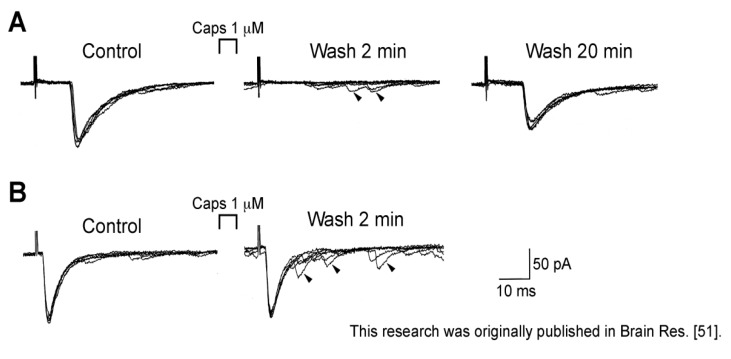
Caps (1 µM) superfused for 0.5 min inhibited monosynaptically-evoked primary-afferent C-fiber but not Aδ-fiber EPSCs in rat SG neurons. In each of (**A**) and (**B**), left, middle and right traces are ones before Caps treatment, 2 and 20 min after Caps wash, respectively, where six superimposed traces are shown. Note an increase in the frequency of sEPSC, some of which are shown by arrows, in the middle traces of (**A**) and (**B**). V_H_ = −70 mV. Modified from [51] with permission of Elsevier Science.

Different from spontaneous excitatory transmission, dorsal root-evoked monosynaptic glutamatergic transmission in SG neurons was inhibited by capsaicin (1 µM). This inhibition was seen for primary-afferent C-fiber but not Aδ-fiber EPSCs (Figure 3). This action was thought to be presynaptic in origin, because Aδ-fiber EPSC amplitude, *i.e.*, AMPA-receptor response, were unaffected by capsaicin. This presynaptic action would be due to either an inhibitory action of capsaicin on voltage-gated Ca^2+^ channels in nerve terminals, a membrane depolarization of C-fiber terminals by capsaicin (such as presynaptic inhibition mediated by GABA_A_-receptor activation; see [48]) or its inhibitory action on nerve conduction (see below). Consistent with the first idea, Bleakman *et al.* [49] have reported a depression by capsaicin of Ca^2+^-channel currents in cultured rat DRG neurons. If this is the case, then this mechanism may have overridden a potentiating action of evoked transmitter release resulting from a capsaicin-induced increase in intraterminal Ca^2+^ concentration leading to an enhancement of spontaneous transmitter release. Urbán and Dray [50] also have reported an inhibitory action of capsaicin on excitatory transmissions elicited in immature mouse dorsal horn neurons by stimulating the dorsal root. As with spontaneous inhibitory transmissions, dorsal root-evoked GABAergic and glycinergic IPSC amplitudes were not affected by capsaicin (1 µM; [51]).

The sEPSC frequency increase produced by capsaicin was accompanied by an averaged inward current of 33 pA. This inward current varied in duration and amplitude among the neurons examined (compare chart recordings in Figure 2B,F). Since primary-afferent C-fibers were known to release a variety of neuroactive peptides including substance P in the dorsal horn ([52,53,54]; for review see [7]), it was likely that the capsaicin-induced inward current was mediated by substance P [50,55]. However, CNQX (10 µM) and TTX (1 µM) but not an NMDA-receptor antagonist dl-2-amino-5-phosphonovaleric acid (APV; 50 µM) and an NK-1-receptor antagonist L-732,138 (1 µM) inhibited the capsaicin current. Moreover, this capsaicin current was not abolished by an intracellular dialysis with GDP-β-S (1 mM), which inhibited a baclofen (10 µM) response mediated by G-protein-coupled GABA_B_ receptors. These results indicate that the capsaicin activity is mediated through the activation of C-fibers by non-NMDA receptors and also by neurotransmitter receptors other than NK1 [56].

**Figure 4:**
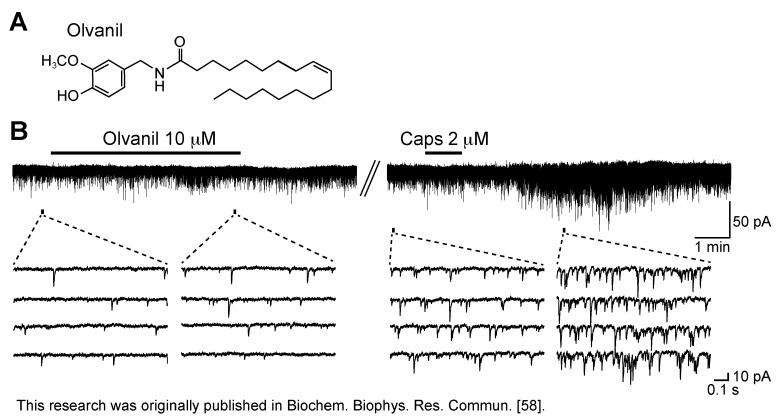
Caps (2 µM) but not olvanil (10 µM) enhances spontaneous excitatory transmission in rat SG neurons. (**A**) The chemical structure of olvanil; (**B**) Recordings of sEPSCs in the absence and presence of olvanil (superfused for 5 min) or Caps (1 min) in the same neuron. The right recording was obtained 20 min after the left one. V_H_ = −70 mV. Modified from [58] with permission of Elsevier Science.

When the effect of the synthetic oleic acid homolog of capsaicin, olvanil (*N*-oleolylvanillylamine; 10 µM; Figure 4A), which activated TRPV1 channels in the cell body of primary-afferent neuron [57], was examined, this drug superfused for 5 min hardly affected spontaneous excitatory transmission in SG neurons. This olvanil concentration was maximal in activating TRPV1 channels in the cell body [57]. In SG neurons insensitive to olvanil, capsaicin (2 µM) superfused for 1 min markedly increased sEPSC frequency (Figure 4B; [58]). This result suggests that there may be a difference in property between the peripheral and central TRP channels.

### 3.2. Action of Resiniferatoxin

**Figure 5:**
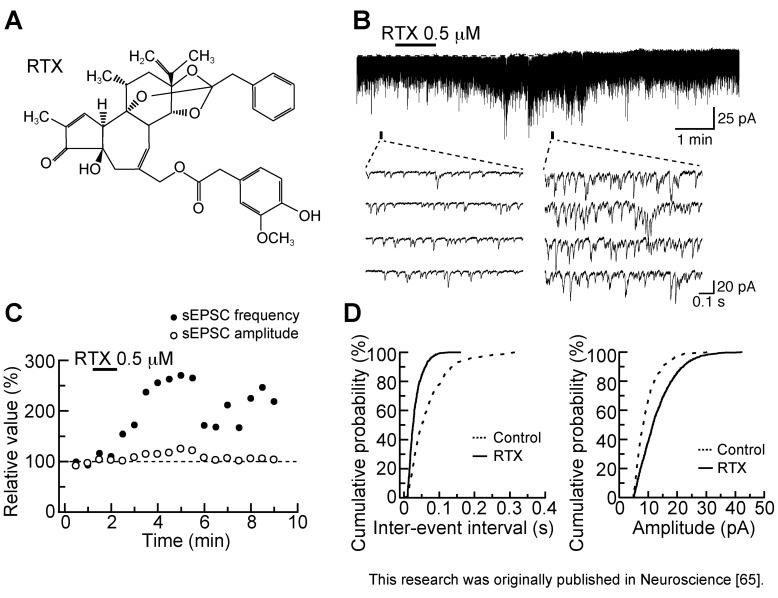
Resiniferatoxin (RTX; 0.5 µM) superfused for 1 min increases the frequency of sEPSC with a small increase in its amplitude in rat SG neurons. (**A**) The chemical structure of RTX; (**B**) Recordings of sEPSCs in the absence and presence of RTX. RTX produces a small peak inward current of 3.5 pA; (**C**) Changes in sEPSC frequency and amplitude (closed and open circles, respectively) were measured every 0.5 min (frequency and amplitude before RTX application: 15.0 Hz and 12.1 pA, respectively); (**D**) Cumulative histograms of the amplitude and inter-event interval of sEPSC before RTX application (Control; dotted line) and under the action of RTX (continuous line). The histograms were examined for 0.5 min in the control (435 sEPSC events) and 3 min after RTX washout (947 sEPSC events). RTX shifted the inter-event interval and amplitude distributions to shorter and larger ones, respectively (*p* < 0.01; Kolmogorov-Smirnov test); (**B**–**D**) were obtained from the same neuron. V_H_ = −70 mV. Modified from [65] with permission of Elsevier Science.

Resiniferatoxin (RTX; Figure 5A) is an ultrapotent TRPV1 agonist, a capsaicin analog isolated from the dried latex of the cactus-like plant, *Euphorbia resinifera* [59,60,61]. [^3^H]RTX is widely used to examine the distribution of TRPV1 channels in the nervous system [62,63]. RTX is also useful in treating disorders, such as neuropathic pain and lower urinary tract dysfunction that involve excessive TRPV1 activity [64].

Bath-applied RTX (0.5 µM) for 1 min enhanced spontaneous excitatory transmission in SG neurons, as seen in Figure 5B. The sEPSC frequency increased gradually over time, peaking around 4 min after RTX addition; this facilitation was accompanied by a small increase in sEPSC amplitude (see Figure 5C). The sEPSC frequency increase was about 136%. This increase in sEPSC frequency did not subside for at least 10 min after RTX washout. With respect to cumulative histograms, RTX significantly increased the proportion of sEPSCs with a shorter inter-event interval and a larger amplitude (Figure 5D; [65]). The sEPSC amplitude increase would be due to highly synchronized multivesicular release of l-glutamate, as shown in ionotropic ATP receptor P2X activation at glutamatergic terminals in the brainstem [66]. As seen in capsaicin actions, a second RTX application 1 or 2 h later did not affect excitatory transmission.

**Figure 6:**
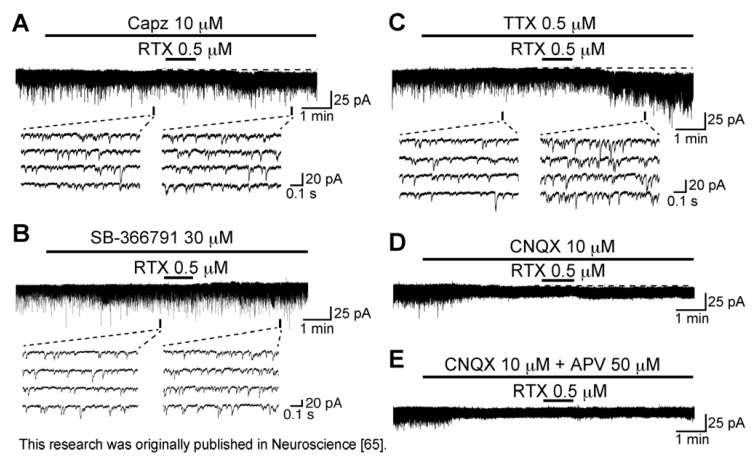
RTX (0.5 µM) enhances spontaneous excitatory transmission by directly activating TRPV1 channels in the rat SG. (**A**–**E**) Recordings in the absence and presence of RTX in Krebs solution containing Capz (10 µM; (**A**)), SB-366791 (30 µM; (**B**)), TTX (0.5 µM; (**C**)), CNQX (10 µM; (**D**)) or both CNQX (10 µM) and an NMDA-receptor antagonist APV (50 µM; (**E**)). V_H_ = −70 mV. Modified from [65] with permission of Elsevier Science.

RTX concentration-dependently increased sEPSC frequency with the effective concentration producing half-maximal response (EC_50_) of 0.21 µM [65]. This value was less than the amount (1 µM; [37]) needed for capsaicin to increase sEPSC frequency in SG neurons, being consistent with a higher affinity (by three to four orders of magnitude) of RTX than capsaicin for TRPV1 channels [67,68]. As seen in capsaicin activity, the ability of RTX to increase sEPSC frequency and amplitude was blocked by capsazepine (10 µM; Figure 6A); a similar inhibition was also seen by another TRPV1 antagonist, SB-366791 (30 µM, [69]; Figure 6B), indicating TRPV1 involvement. This idea was supported by the observation that capsaicin inhibited the effects of RTX on excitatory transmission and *vice versa*, *i.e.*, cross-desensitization [65]. A similar cross-desensitization of RTX and capsaicin activity occurs for the release of substance P or calcitonin gene-related peptide from the central terminals of primary-afferent neurons in the rat spinal cord [67]. As seen from Figure 6C, the sEPSC frequency increase produced by RTX was resistant to TTX, a result similar to that of capsaicin. On the other hand, as different from capsaicin, RTX induced inward currents in a manner resistant to TTX, indicating that RTX may have activated TRPV1 channels in postsynaptic neurons. TRPV1 channels exist in both pre- and postsynaptic neurons in the superficial laminae of the rat spinal dorsal horn [43]. However, RTX-induced inward current was blocked by CNQX (10 µM) and APV (50 µM) treatment (Figure 6D,E), indicating that the inward current resulted from activation of non-NMDA and NMDA receptors by l-glutamate released from the primary-afferent terminals after TRPV1 activation. Consistent with this finding regarding NMDA receptors, several subtypes of NMDA receptors are found in SG neurons [70]. The NMDA receptor-mediated postsynaptic current can be evoked by the application of NMDA and by focal or dorsal root stimulation at −70 mV [71,72]. Even if RTX binds to the same TRPV1 site as capsaicin (see [63,73]), their activities on excitatory transmission may be different from each other. RTX but not capsaicin mobilized Ca^2+^ from inositol 1,4,5-trisphosphate (IP_3_)-sensitive Ca^2+^ stores in TRPV1-transfected human embryonic kidney (HEK) 293 cells [74]. Understanding the differences in inward currents produced by RTX and capsaicin in SG neurons requires further work.

### 3.3. Action of Piperine

**Figure 7:**
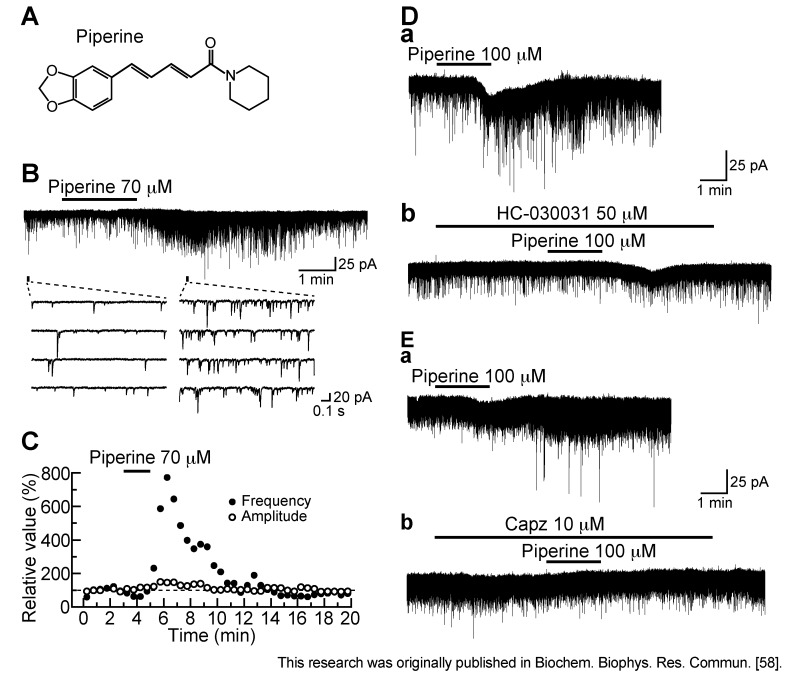
Effect of piperine on spontaneous excitatory transmission in rat SG neurons. (**A**) The chemical structure of piperine; (**B**) Recordings of sEPSCs in the absence and presence of piperine (70 µM); (**C**) sEPSC frequency and amplitude (closed and open circles, respectively) under the action of piperine, relative to those before its superfusion (frequency and amplitude: 3.6 Hz and 13.4 pA, respectively). They were measured every 0.5 min; (**B**) and (**C**) were obtained from the same neuron; (**D**,**E**) Chart recordings showing the action of piperine (100 µM) on excitatory transmission in the absence (**a**) and presence (**b**) of a TRPA1 antagonist HC-030031 (50 µM; **D**) or Capz (10 µM; **E**). V_H_ = −70 mV. Modified from [58] with permission of Elsevier Science.

Piperine (1-peperoylpiperidine; Figure 7A; [75]) is the pungent component of black pepper and has an ability to activate TRPV1 channels in the cell body of primary-afferent neuron [76]. As seen from Figure 7B, piperine (70 µM) superfused for 2 min enhanced spontaneous excitatory transmission in SG neurons. The sEPSC frequency increased gradually over time, peaking around 3 min after piperine addition; this facilitation was accompanied by a minimal increase in sEPSC amplitude (Figure 7C). The sEPSC frequency increase produced by piperine was repeated and concentration-dependent with the EC_50_ value of 52.3 µM [58]. This value was similar to that (35 µM) for activating TRPV1 channels in rat trigeminal ganglion neurons [76] or that (37.9 µM) of human cloned TRPV1 channels expressed in HEK 293 cells [77]. Under the pretreatment with capsazepine (10 µM) or a TRPA1 antagonist HC-030031 (50 µM; [78]), the facilitatory effect of piperine (100 µM) on sEPSC frequency was reduced in extent (Figure 7D,E). The inhibitory action of HC-030031 on piperine response was suggested to be due to a nonspecific blockade of TRPV1 channel, because HC-030031 was reported to also act as a TRPV1 antagonist [79]. Consistent with this idea, Okumura *et al.* [80] have reported that piperine activates, not only human cloned TRPV1, but also TRPA1 channels expressed in HEK 293 cells, albeit the TRPV1 channel has EC_50_ value (0.6 µM) smaller by 50-fold than that (29.7 µM) of the TRPA1 channel.

Although both piperine and capsaicin activated TRPV1 channels, the former but not latter action was repeated. The difference in recovery from desensitization between piperine and capsaicin actions may be attributed to a distinction in their binding to TRPV1 channels. This idea is supported by the observation that there is a difference in the rate of the onset of desensitization and its degree between TRPV1 channel activations produced by capsaicin and piperine in rat trigeminal ganglion neurons [76] or human HEK 293 cells [77].

### 3.4. Action of Allyl Isothiocyanate

**Figure 8:**
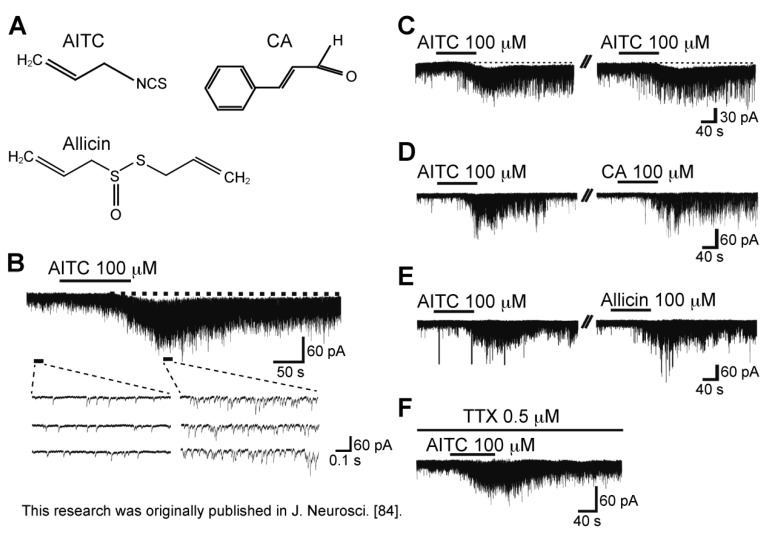
Actions of a TRPA1 agonist allyl isothiocyanate (AITC) and related substances (cinnamaldehyde (CA) and allicin) on spontaneous excitatory transmission in rat SG neurons. (**A**) The chemical structures of AITC, CA and allicin; (**B**) Recording of sEPSCs before and during the action of AITC (100 μM). Note a slow inward current that is accompanied by increases in sEPSC frequency and amplitude; (**C**) When AITC (100 μM) was applied repeatedly at 20 min intervals, it produced similar increases in sEPSC frequency and amplitude and induced an inward current having a similar amplitude; (**D**,**E**) CA (**D**) or allicin (each 100 μM); (**E**) increased sEPSC frequency and amplitude in a neuron in which AITC increased sEPSC frequency and amplitude. In each of (**C**–**E**), the right recording was obtained 15 min after the left one; (**F**) The sEPSC frequency and amplitude increase produced by AITC persisted in the presence of TTX (0.5 μM). V_H_ = −70 mV. Modified from [84] with permission of Journal of Neuroscience.

Bath-applied allyl isothiocyanate (AITC, Figure 8A; the pungent principal in mustard oil or wasabi; 100 μM) for 2 min resulted in an increase in the frequency and amplitude of sEPSC in SG neurons; this action was often accompanied by a slow inward current, as seen in Figure 8B. The sEPSC frequency increase averaged to be 202%. When AITC (100 µM) was applied repeatedly at 20 min intervals, it induced a similar increase in sEPSC frequency and amplitude (Figure 8C), an observation different from those of capsaicin and RTX. A similar action was seen by pungent natural compounds having an ability to activate TRPA1 channels, cinnamaldehyde (CA; 100 μM) and allicin (100 μM; Figure 8A; for similar observations see [81,82]), which are contained in cinnamon oil, ginger and garlic [26,83]. There was no interaction between the AITC action and the CA or allicin action, when examined by their repeated applications (Figure 8D,E; [84]). TTX (0.5 µM) did not affect sEPSC frequency increases produced by AITC, as well as capsaicin and RTX (Figure 8F), indicating a direct action of AITC. A similar action of AITC has been reported in the superficial medullary dorsal horn [85].

**Figure 9:**
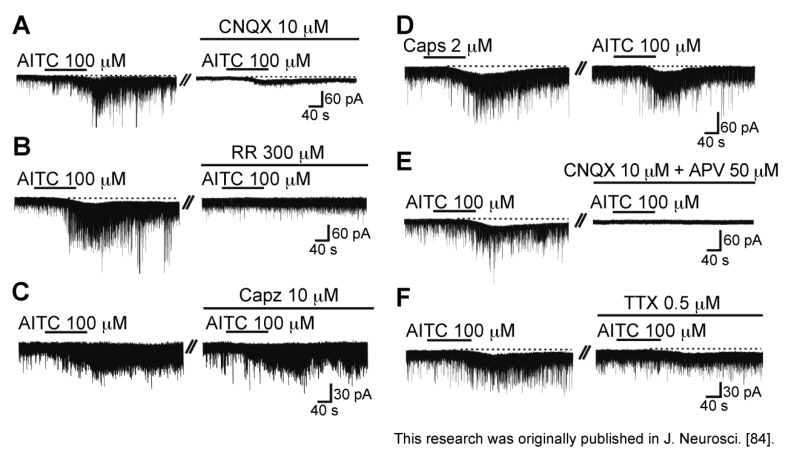
Characterization of the sEPSC frequency and amplitude increase and inward current produced by AITC. (**A**–**C**,**E**,**F**) Action of AITC (100 μM) on sEPSCs in the absence (left) and presence (right) of CNQX (10 μM; (**A**)), a non-selective TRP antagonist ruthenium red (RR; 300 μM; (**B**)), Capz (10 μM; (**C**)), both of CNQX (10 μM) and APV (50 μM; (**E**)), or TTX (0.5 μM; (**F**)); (**D**) Actions of Caps (2 μM) and AITC (100 μM) on sEPSCs in the same neuron. In each of (**A**–**F**), the right recording was obtained 15 min after the left one. V_H_ = −70 mV. Modified from [84] with permission of Journal of Neuroscience.

As seen in the facilitatory action of capsaicin, CNQX (10 μM) suppressed sEPSCs not only before AITC superfusion but also under the action of AITC (Figure 9A), indicating that AITC caused a robust l-glutamate release onto SG neurons. As seen in Figure 9B, the AITC-induced increases in sEPSC frequency and amplitude were suppressed by a non-selective TRP antagonist ruthenium red (300 μM). On the other hand, the AITC activities were not affected by capsazepine (10 μM; Figure 9C), and there was no interaction between the AITC and capsaicin actions (Figure 9D), indicating an involvement of TRP channels other than TRPV1 channel, *i.e.*, TRPA1 channel [84]. Consistent with this idea, rat DRG neurons express TRPA1 mRNAs and proteins [22,29,86]. The AITC-induced sEPSC frequency increase disappeared in a nominally Ca^2+^-free solution, indicating that this increase is due to a high Ca^2+^ permeability of TRPA1 channels [87] and voltage-gated Ca^2+^ channel opening in nerve terminals. The latter mechanism appeared to be not involved in the AITC activity, because AITC increased sEPSC frequency in the presence of a voltage-gated Ca^2+^-channel blocker La^3+^ (30 µM; [84,88]).

With respect to the AITC-induced inward current, this amplitude was reduced by ruthenium red (300 μM; Figure 9B) and APV (50 µM; Figure 9E), but not CNQX (10 µM) and TTX (0.5 µM; Figure 9A,F). These results suggest that the AITC current is mediated by the activation of postsynaptic NMDA receptors in SG neurons. These results suggest that inward currents produced by TRPV1 agonists (capsaicin and RTX) and TRPA1 agonist (AITC) may be different in origin from each other.

**Figure 10:**
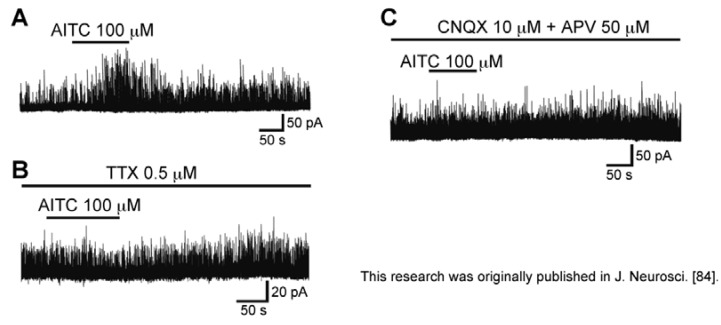
Action of AITC (100 μM) on spontaneous inhibitory transmission in rat SG neurons. (**A**) Chart recording of spontaneous inhibitory postsynaptic currents (IPSCs) before and during the action of AITC; (**B**,**C**) Chart recording of spontaneous IPSCs before and during the action of AITC in the presence of TTX (0.5 µM; (**B**)) or a mixture of CNQX (10 μM) and APV (50 μM; (**C**)). V_H_ = 0 mV. Modified from [84] with permission of Journal of Neuroscience.

As different from capsaicin, AITC (100 µM) increased GABAergic and/or glycinergic spontaneous IPSC frequency and amplitude in SG neurons (Figure 10A). This AITC-induced enhancement of inhibitory transmission was abolished in the presence of TTX (0.5 µM), as well as in a mixture of CNQX (10 µM) and APV (50 µM; Figure 10B,C). Therefore, TRPA1 channels were suggested to be localized, not only at presynaptic terminals on SG neurons, but also in primary afferent fibers innervating onto spinal inhibitory interneurons which make synapses with SG neurons [84]. Consistent with this idea, double patch-clamp recordings from SG neurons have revealed that inhibitory connections are present between two kinds of SG neurons [89]. Both of the presynaptic islet cell and postsynaptic central neuron receive monosynaptic inputs from different C-fibers, where the islet cell is an inhibitory interneuron and postsynaptic to the central neuron [89]. The input to the presynaptic islet cell is from larger-diameter, more rapidly conducting C-fibers than those projecting to the postsynaptic central neuron (see [84]). Although AITC and CA have an ability to activate TRPA1 channels, Uta *et al.* [81] have reported that CA does not affect inhibitory transmissions in SG neurons. Each of AITC and CA may activate TRPA1 channels located on different types of primary-afferent neurons.

### 3.5. Action of Eugenol

**Figure 11:**
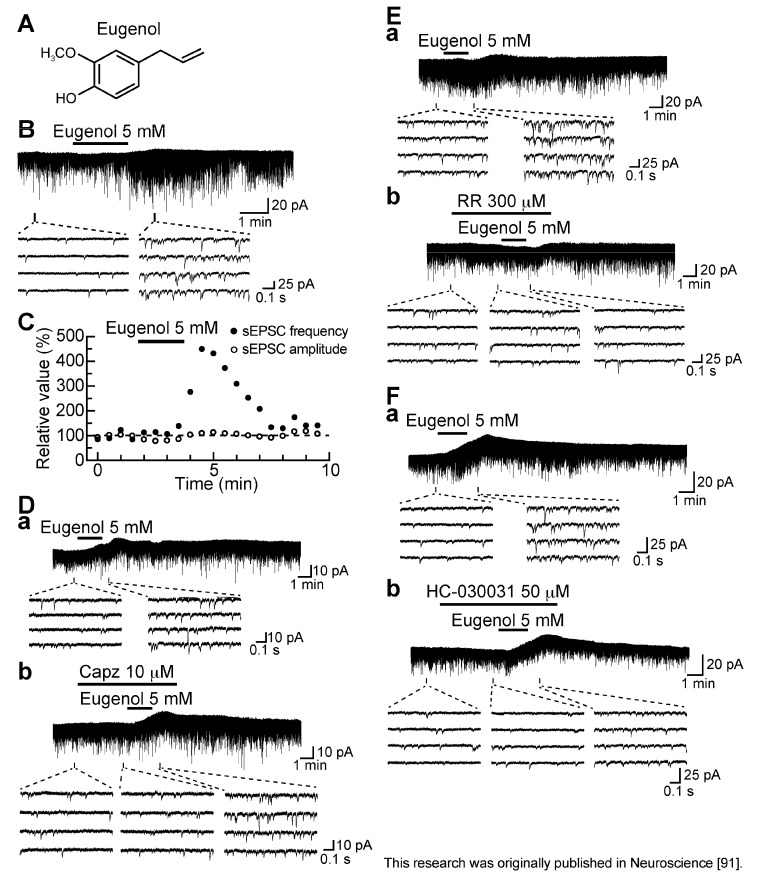
Eugenol (5 mM) enhances spontaneous excitatory transmission in rat SG neurons by TRPA1 but not TRPV1 activation. (**A**) The chemical structure of eugenol; (**B**) Recordings of sEPSCs in the absence and presence of eugenol. Eugenol produces a peak outward current of 6.4 pA; (**C**) Time courses of changes in sEPSC frequency and amplitude (closed and open circles, respectively) during the action of eugenol, relative to those before its superfusion (frequency and amplitude: 4.33 Hz and 13.8 pA, respectively), where they were measured every 0.5 min. (**B**,**C**) were obtained from the same neuron; (**D**–**F**) Recordings of sEPSCs under the action of eugenol in the absence (**a**) and presence (**b**) of Capz (10 µM; **D**), RR (300 µM; **E**) or HC-030031 (50 µM; **F**). V_H_ = −70 mV. Modified from [91] with permission of Elsevier Science.

Eugenol [2-methoxy-4-(2-propenyl) phenol, a vanilloid compound; Figure 11A] is an aromatic molecule contained in several plants including clove and bay leaves, and has been widely used as an analgesic to treat toothache [90]. As seen in Figure 11B, superfusing eugenol (5 mM) for 2 min enhanced spontaneous excitatory transmission in SG neurons. The frequency of sEPSC increased gradually over time, peaking around 2.5 min after eugenol addition; this facilitation was accompanied by a small increase in its amplitude (Figure 11C). This frequency increase was on average about 461%. As seen in the AITC and capsaicin actions, the eugenol action was not affected by TTX (0.5 µM). Eugenol as well as AITC repeatedly increased sEPSC frequency, a result different from those of capsaicin and RTX. The sEPSC frequency increase was concentration-dependent with the EC_50_ value of 3.8 mM [91]. Since eugenol reportedly activated TRPV1 channels, which were cloned [92] and were expressed in rat primary-afferent neurons ([93] albeit less effective than capsaicin), we examined how the spontaneous excitatory transmission enhancement by eugenol is affected by capsazepine. Capsazepine (10 µM) did not block the ability of eugenol to increase sEPSC frequency and amplitude (Figure 11D).

On the contrary, ruthenium red (300 µM) and HC-030031 (50 µM) inhibited the facilitatory effect of eugenol on excitatory transmission (Figure 11E,F; [91]), indicating an involvement of TRPA1 but not TRPV1 channels. Considering that half-maximal inhibitory concentration (IC_50_) for HC-030031 in inhibiting human TRPA1 activation by AITC is 0.7 µM [78], the concentration of HC-030031 (50 µM) used in this study may have been quite high. However, a high concentration of HC-030031, such as 100 µM, appeared to be necessary to inhibit TRPA1 activation in the rat spinal or medullary dorsal horn [81,94].

The sEPSC frequency increase produced by eugenol would be due to an increase in Ca^2+^ entry from extracellular solution, because the TRPA1 channel is a non-selective cation channel permeable to Ca^2+^ ions [87]. Consistent with this idea, eugenol produced Ca^2+^-permeable currents in cultured rat DRG neurons [95].

Different from the above-mentioned TRP agonists, eugenol produced an outward but not inward current, which was resistant to capsazepine (10 µM), ruthenium red (300 µM) and HC-030031 (50 µM), as noted from Figure 11D–F [91], indicating no involvement of TRP channels. This outward current (hyperpolarizing) effect may explain antinociception produced by eugenol, because endogenous analgesics such as opioids, nociceptin, serotonin, norepinephrine, adenosine and galanin hyperpolarize the membrane of SG neurons (for example see [9,14,96,97,98,99]; for review see [15]). Ohkubo and Shibata [100] have shown that eugenol inhibits formalin-induced nociceptive responses in mice. Furthermore, eugenol alleviated neuropathic pain in sciatic nerve ligation rat models [101]. A similar alleviation was produced by the oral administration of eugenol [102]. The antinociceptive effect of eugenol was, however, sensitive to capsazepine [100], an observation being inconsistent with our result that the eugenol-induced hyperpolarization was not mediated by TRPV1 and TRPA1 channels. This issue remains to be addressed.

### 3.6. Action of Zingerone

**Figure 12:**
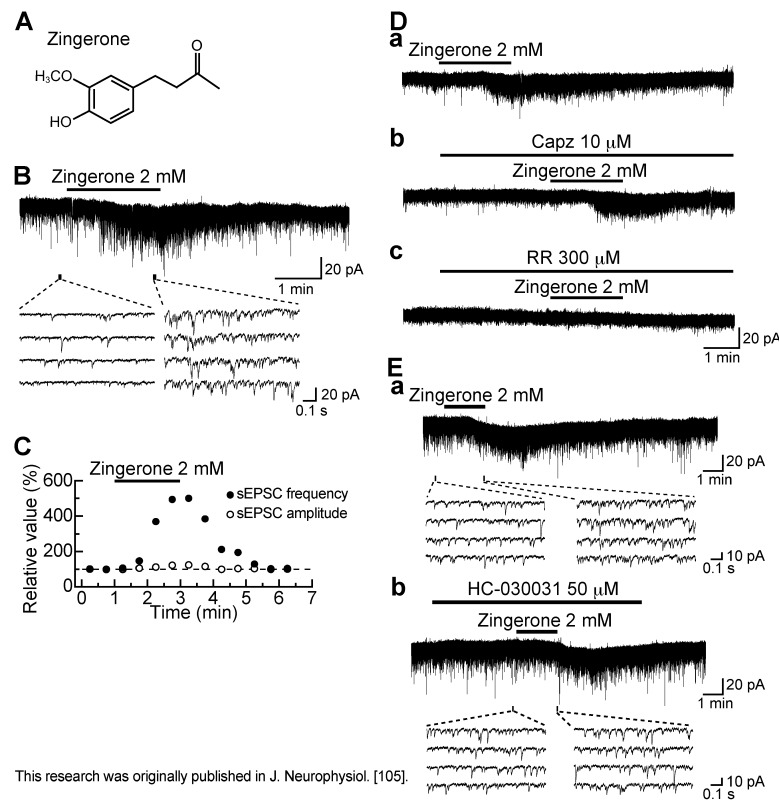
Zingerone (2 mM) enhances spontaneous excitatory transmission in rat SG neurons by TRPA1 but not TRPV1 activation. (**A**) The chemical structure of zingerone; (**B**) Recordings of sEPSCs in the absence and presence of zingerone; (**C**) Time courses of changes in sEPSC frequency and amplitude (closed and open circles, respectively) during the action of zingerone, relative to those before its superfusion; (**D**,**E**) Recordings of sEPSCs under the action of zingerone in the absence (**a**) and presence (**b**,**c**) of Capz (10 µM; **D**), RR (300 µM; **D**) or HC-030031 (50 µM; **E**). (**Da**), (**Db**) and (**Dc**), or (**Ea**) and (**Eb**) were obtained from the same neuron. V_H_ = −70 mV. Modified from [105] with permission of Journal of Neurophysiology.

Zingerone (4-(4-hydroxy-3-methoxyphenyl)-2-butanone; Figure 12A) is a pungent component of ginger, rhizomes of *Zingiber officinale* Roscoe [103], which is a vanilloid compound and activates TRPV1 channels in the cell body of the primary-afferent neuron [76,104]. Zingerone (2 mM) superfused for 2 min enhanced spontaneous excitatory transmission in SG neurons (Figure 12B). The frequency of sEPSC increased gradually over time, peaking around 2.5 min after the onset of zingerone addition, and this facilitation was accompanied by a small increase in its amplitude (Figure 12C). This frequency increase averaged to be about 260%. As with eugenol, the activity of zingerone was repeated and resistant to TTX (0.5 µM). The sEPSC frequency increase was concentration-dependent with the EC_50_ value of 1.3 mM [105]. The sensitivities of the zingerone activity to TRP antagonists were similar to those of the eugenol activity. The sEPSC frequency increase produced by zingerone (2 mM) was resistant to capsazepine (10 µM) while being sensitive to ruthenium red (300 µM) or HC-030031 (50 µM; Figure 12D,E), indicating an involvement of TRPA1 channels. Consistent with this idea, AITC (100 µM) inhibited the facilitatory action of zingerone (2 mM) on excitatory transmission [105].

Since the sEPSC frequency increase produced by zingerone as well as AITC was resistant to La^3+^ (30 µM), it was unlikely that this increase was mediated by voltage-gated Ca^2+^-channel activation following a membrane depolarization produced by zingerone, resulting in intraterminal Ca^2+^ concentration increase [105]. TRPA1 channel is permeable to Ca^2+^ [27,87] and, thus, this channel activation would result in Ca^2+^ entry from external solution, followed by an increase in intraterminal Ca^2+^ concentration and then sEPSC frequency. However, different from AITC (see above), the activity of zingerone was not depressed in Ca^2+^-free solution [105]. TRPA1 activations by zingerone and AITC may be distinct from each other in mode such as desensitization and intracellular Ca^2+^ mobilization. As shown in rat primary-afferent neurons, TRPA1 desensitization in nerve terminals may be decreased in extent in Ca^2+^-free solution [106,107]. The decrease in the desensitization of zingerone activity may result in TRPA1 activity increase, which overrides the decrease in Ca^2+^ entry through TRPA1 channels in Ca^2+^-free solution. It is possible that desensitization is distinct in extent between zingerone- and AITC-activated TRPA1 responses, because agonist-dependent desensitization has been demonstrated for many types of ligand-gated channel including TRPA1 and TRPV1 channels [76,107]. Alternatively, it is likely that intraterminal Ca^2+^ level increases produced by zingerone and AITC are distinct from each other, because a Ca^2+^ permeability of TRPA1 channel alters in extent, depending on agonists involved in its activation [87,108]. Intraterminal Ca^2+^ level increase by TRPA1 activation may be due to not only Ca^2+^ entry from external solution but also Ca^2+^ release from intracellular stores. Although intracellular Ca^2+^ level rise is known to activate phospholipase C, resulting in the production of IP_3_ [109], IP_3_-sensitive Ca^2+^ stores do not appear to be involved in the zingerone activity, because this activity is not inhibited but rather facilitated by an IP_3_-induced Ca^2+^-release inhibitor 2-aminoethoxydiphenyl borate (2-APB; [105,110]). On the other hand, Ca^2+^-induced Ca^2+^-release mechanisms appeared to be involved in the presynaptic action of zingerone, because a Ca^2+^-induced Ca^2+^-release inhibitor dantrolene [111] reduced the extent of sEPSC frequency increase produced by zingerone [105]. Taken together, a small Ca^2+^ influx through TRPA1 channels activated by zingerone in nominally Ca^2+^-free solution may mobilize Ca^2+^ from intracellular stores, probably through Ca^2+^-induced Ca^2+^-release mechanisms, leading to sEPSC frequency increase. The difference in Ca^2+^ sensitivity between TRPA1 activations by zingerone and AITC remains to be examined.

Since TRPV1 and TRPA1 activations inhibited monosynaptically-evoked excitatory transmission in adult rat SG neurons [51,81], we examined how zingerone (2 mM) affects monosynaptic Aδ-fiber and C-fiber EPSCs in the SG neurons. Each of the peak amplitudes of monosynaptic Aδ-fiber and C-fiber EPSCs evoked by stimulating the dorsal root was reduced by zingerone in a reversible manner with a similar extent, an observation different from the inhibitory actions of capsaicin and CA, where C-fiber EPSCs were much more sensitive to the drugs than Aδ-fiber ones [51,81]. Zingerone activity was also distinct from those of capsaicin and CA in their actions on spontaneous inhibitory transmissions. Like AITC, zingerone enhanced spontaneous GABAergic transmission in a manner sensitive to TTX [105], but capsaicin and CA had no effects on inhibitory transmissions [37,51,81]. Each of the TRP agonists appeared to have different action on monosynaptic primary-afferent and spontaneous inhibitory transmissions.

The zingerone-induced inward current was unaffected by CNQX and APV [105], an observation different from that of AITC-induced one (see above). On the other hand, ruthenium red or HC-030031 reduced the peak amplitude of the zingerone current [105], suggesting that TRPA1 channels may be expressed in SG neurons. This is consistent with the presence of TRPA1 immunoreactivity in the rat superficial dorsal horn [86]. It remains to be examined whether TRPA1 channels exist in SG neurons. The difference in property between TRP channels in the central terminal and cell body of primary-afferent neuron, as seen in the zingerone and eugenol actions, has been noted for actions of a local anesthetic. Lidocaine, which acted on TRPV1 channels [112] and by a less extent on TRPA1 channels [113] in the cell body of DRG neuron, activated TRPA1 but not TRPV1 channels in its central terminal in the SG [114]. Many of DRG neurons co-express TRPA1 and TRPV1 channels [22,29] and a TRPA1-TRPV1 channel complex may be formed on the plasma membrane [115]. This may result in a distinction in property between the peripheral and central TRP channels. Alternatively, there may be TRP splice variants (for example see [116,117]) or TRP channels modulated by second messengers (for example see [118,119,120]), which are distinct in property between the peripheral and central terminals.

### 3.7. Action of Menthol

Since a representative TRPM8 agonist menthol enhanced sEPSC frequency in SG neurons [39,82,121], TRPM8 as well as TRPV1 and TRPA1 activation in the SG appeared to result in the enhancement of spontaneous excitatory transmission. Although TRPM8 channels are expressed in rat DRG neurons, they are not co-expressed with TRPV1 and TRPA1 channels [22,29]. The properties of TRPM8 channels located in primary-afferent central terminals have not yet been examined thoroughly.

### 3.8. Nerve Conduction Inhibition by TRP Agonists

TRP agonists have an ability not only to modulate synaptic transmission but also to inhibit nerve conduction, *i.e.*, voltage-gated ion channels. For example, capsaicin is known to block the conduction of impulses in many, but not all, of C fibers contained in rat vagus nerves [122], mouse and rat dorsal roots [123,124], and human sural nerves [125]. Urbán and Dray [124] have also reported that Aδ fibers in mouse (13–18 day-old) dorsal roots may be also sensitive to capsaicin at concentrations of 1–5 µM. Moreover, capsaicin inhibits voltage-gated Na^+^-channels in a TRPV1-dependent [126,127,128] or -independent manner. The latter action has been shown to be due to either a change in lipid bilayer elasticity [129] or a direct action on Na^+^ channels themselves [127,130]. Like capsaicin, RTX inhibited voltage-gated Na^+^ and Ca^2+^ channels [131] and also AP conduction in primary-afferent fibers [132]. Eugenol also inhibited voltage-gated Na^+^ and K^+^ channels without TRPV1 activation [133,134,135]. TRP agonists other than vanilloids also inhibit voltage-gated channels. Haeseler *et al.* [136] have reported that menthol inhibits neuron- and muscle-type voltage-gated Na^+^ channels expressed in heterologous HEK 293 cells. TTX-sensitive Na^+^-channel current amplitudes in immortalized DRG neuron-derived F11 cells were reduced by (+)-menthol [137].

We have examined the actions of TRP agonists on TTX-sensitive and fast-conducting compound APs (CAPs) recorded from the frog sciatic nerve by using the air-gap method [138]. Capsaicin reversibly and concentration-dependently reduced the peak amplitude of the CAP (by 34% at 0.1 mM). Capsazepine did not affect the capsaicin activity, and RTX had no effect on CAPs, indicating no involvement of TRPV1 channels. Capsaicin analogs and other various vanilloids also inhibited CAPs in a concentration-dependent manner. An efficacy sequence of these inhibitions was capsaicin > eugenol > zingerone; olvanil was effective less than capsaicin. Nerve CAP inhibition by eugenol has been reported previously [139,140]. Thus, various vanilloids exhibit CAP inhibition, the extent of which is determined by the property of the side chain bound to the vanillyl group [141]. Menthol also concentration-dependently reduced CAP peak amplitude with the IC_50_ value of 1 mM in a manner resistant to ruthenium red, and a TRPM8 agonist icilin did not affect CAPs, indicating no involvements of TRPM8 channels [142]. Similar CAP inhibitions were also produced by TRPA1 agonists. AITC and CA concentration-dependently reduced the peak amplitude of the CAP with the IC_50_ values of 1.5 and 1.2 mM, respectively, and these activities were resistant to ruthenium red or HC-030031, indicating no involvement of TRPA1 channels [143]. The efficacies of the TRP agonists in inhibiting CAPs were almost comparable to those of local anesthetics such as lidocaine and procaine. Inhibitions of dorsal root-evoked EPSCs produced by TRP agonists at high concentrations in SG neurons may be partly due to their inhibitory actions on nerve conduction.

## 4. Physiological Significance of TRP Channels in the Substantia Gelatinosa

The above-mentioned TRP agonists derived from plants are not ones that act on TRP channels located in the central terminals of DRG neurons under physiological conditions. Therefore, there should be endogenous substances, which activate the central TRP channels. There are several candidates for the substances. For example, endogenous agonists for TRPV1 channels include endocannabinoids and lipoxygenase metabolites, which have structures similar to that of capsaicin which is not produced endogenously ([144,145]; for review see [25,146]). Moreover, there appears to be an endogenous capsaicin-like substance, which is released from inflamed tissues, resulting in the production of nociceptive neural impulses by activating TRPV1 channels [147].

With respect to TRPA1 channels, 3′-carbamoylbiphenyl-3-yl cyclohexylcarbamate (URB597), a potent and systemically active inhibitor of fatty acid amide hydrolase, has been demonstrated to be a synthetic activator [148]. Cruz-Orengo *et al.* [149] have identified as an endogenous TRPA1 agonist a cyclopentane prostaglandin D_2_ metabolite (15-deoxy-Δ^12,14^-prostaglandin J_2_) by using a bioactive lipid library screen. Moreover, as candidates of endogenous TRPA1 agonists there are a cytochrome P450-epoxygenase-derived metabolite (5,6-epoxyeicosatrienoic acid (5,6-EET); [150]) or a 12-lipoxygenase-derived metabolite (hepoxilin A_3_; [151]) of arachidonic acid. As with AITC, 5,6-EET increased sEPSC frequency in SG neurons; this was not accompanied by a change in its amplitude [150]. Inflammatory peptide bradykinin is also a possible endogenous ligand, because TRPA1 channels can be activated by its action on phospholipase C signaling pathways [26,83]. Although bradykinin is considered to be a peripherally acting mediator, bradykinin production in the spinal cord is induced by peripheral inflammation [152]. Furthermore, it has been demonstrated that bradykinin also increases l-glutamate release from primary-afferent central terminals onto SG neurons [152].

There is much evidence showing that TRP channels are involved in nociceptive transmission. For instance, inflammatory hyperalgesia and neuropathic pain develop due to TRPV1 over-expression or ectopic expression in both the central nervous system and peripheral nervous system, in animals and in humans [153,154,155,156]. TRPV1-knockout mice exhibit reduced hyperalgesia in inflammatory pain models [157]. Nerve growth factor, a key mediator of inflammatory pain, upregulates TRPV1 expression in rat DRG neurons through the small GTPase Ras [158]. The TRPV1 channel is expressed in neurons involved in the sensation of cancer pain and is a potential treatment target for this pain (for review see [159]). Selective blockade of TRPV1 channels attenuates bone cancer pain in the mouse [160]. Intrathecal administration of a TRPV1 antagonist AS1928370 inhibited mechanical allodynia in a mouse model of neuropathic pain [161]. Intrathecal administration of RTX in dogs with bone cancer produces a prolonged antinociceptive response [162]. Upregulated TRPV1 expression in primary-afferent fibers occurs in disease states such as inflammatory disease and irritable bowel syndrome [163,164]. Peripheral inflammation upregulates TRPV1 channels involved in enhancing spontaneous excitatory transmission in rat SG neurons [69].

With respect to TRPA1 channels, CA can evoke spontaneous pain and induce mechanical hyperalgesia and cold hypoalgesia when it is delivered to the forearm skin of human volunteers [165]. Moreover, TRPA1 channels are upregulated in rat DRG neurons after peripheral inflammation and nerve injury, and inflammation- and nerve injury-induced cold hyperalgesia is mediated by TRPA1 but not TRPM8 channels [166]. Alternatively, TRPA1 channels are over-expressed in mouse spinal cord and DRG following peripheral inflammation induced by intraplantar injection of complete Freund’s adjuvant; intrathecal administration of a TRPA1 antagonist reverses hyperalgesia in mouse models of neuropathic pain [167]. Such an antinociceptive effect produced by intrathecally-administered TRPA1 antagonist is observed under various pain hypersensitivity conditions, for example, after rapid eye movement sleep deprivation, spinal nerve ligation, and the intraplantar application of formalin or capsaicin [168].

## 5. Conclusions

TRP channels in the peripheral terminal of DRG neuron receive nociceptive stimuli given to the periphery while TRP channels in the central terminal of the neuron are involved in the modulation of nociceptive transmission. Their actions are due to membrane depolarization and spontaneous l-glutamate release enhancement, respectively. Although the TRP channels are synthesized in the cell body of DRG neuron and then are transferred to the peripheral and central terminals of the neuron by axonal transport, the peripheral and central TRP channels appear to be different in property from each other.
